# Machine Learning Approaches for Predicting Hypertension and Its Associated Factors Using Population-Level Data From Three South Asian Countries

**DOI:** 10.3389/fcvm.2022.839379

**Published:** 2022-03-31

**Authors:** Sheikh Mohammed Shariful Islam, Ashis Talukder, Md. Abdul Awal, Md. Muhammad Umer Siddiqui, Md. Martuza Ahamad, Benojir Ahammed, Lal B. Rawal, Roohallah Alizadehsani, Jemal Abawajy, Liliana Laranjo, Clara K. Chow, Ralph Maddison

**Affiliations:** ^1^Institute for Physical Activity and Nutrition, Faculty of Health, Deakin University, Melbourne, VIC, Australia; ^2^Statistics Discipline, Khulna University, Khulna, Bangladesh; ^3^Electronics and Communication Engineering Discipline, Khulna University, Khulna, Bangladesh; ^4^Department of Hospital Medicine, Thomas Jefferson University, Philadelphia, PA, United States; ^5^Department of Computer Science and Engineering, Bangabandhu Sheikh Mujibur Rahman Science and Technology University, Gopalganj, Bangladesh; ^6^School of Health Medical and Applied Sciences, Central Queensland University, Sydney, NSW, Australia; ^7^Institute for Intelligent Systems Research and Innovation, Deakin University, Geelong, VIC, Australia; ^8^School of Information Technology, Deakin University, Geelong, VIC, Australia; ^9^Faculty of Medicine and Health, Westmead Applied Research Centre, The University of Sydney, Sydney, NSW, Australia

**Keywords:** Demographic and Health Survey, blood pressure, algorithms, risk factors, South Asia, artificial intelligence, cardiovascular diseases

## Abstract

**Background:**

Hypertension is the most common modifiable risk factor for cardiovascular diseases in South Asia. Machine learning (ML) models have been shown to outperform clinical risk predictions compared to statistical methods, but studies using ML to predict hypertension at the population level are lacking. This study used ML approaches in a dataset of three South Asian countries to predict hypertension and its associated factors and compared the model's performances.

**Methods:**

We conducted a retrospective study using ML analyses to detect hypertension using population-based surveys. We created a single dataset by harmonizing individual-level data from the most recent nationally representative Demographic and Health Survey in Bangladesh, Nepal, and India. The variables included blood pressure (BP), sociodemographic and economic factors, height, weight, hemoglobin, and random blood glucose. Hypertension was defined based on JNC-7 criteria. We applied six common ML-based classifiers: decision tree (DT), random forest (RF), gradient boosting machine (GBM), extreme gradient boosting (XGBoost), logistic regression (LR), and linear discriminant analysis (LDA) to predict hypertension and its risk factors.

**Results:**

Of the 8,18,603 participants, 82,748 (10.11%) had hypertension. ML models showed that significant factors for hypertension were age and BMI. Ever measured BP, education, taking medicine to lower BP, and doctor's perception of high BP was also significant but comparatively lower than age and BMI. XGBoost, GBM, LR, and LDA showed the highest accuracy score of 90%, RF and DT achieved 89 and 83%, respectively, to predict hypertension. DT achieved the precision value of 91%, and the rest performed with 90%. XGBoost, GBM, LR, and LDA achieved a recall value of 100%, RF scored 99%, and DT scored 90%. In F1-score, XGBoost, GBM, LR, and LDA scored 95%, while RF scored 94%, and DT scored 90%. All the algorithms performed with good and small log loss values <6%.

**Conclusion:**

ML models performed well to predict hypertension and its associated factors in South Asians. When employed on an open-source platform, these models are scalable to millions of people and might help individuals self-screen for hypertension at an early stage. Future studies incorporating biochemical markers are needed to improve the ML algorithms and evaluate them in real life.

## Introduction

Hypertension is the leading cause of cardiovascular disease attributing to 8.5 million deaths globally, with 88% deaths in low-income and middle-income countries (1, 2). In South Asia, the prevalence of hypertension has been increasing primarily due to increased access to unhealthy foods, sedentary lifestyles, and rural-urban migration (3, 4). South Asia also has the lowest rates of hypertension detection, treatment, and control, with little improvement in these outcomes over the past three decades (2). Many people with hypertension remain largely undetected in South Asia due to decreased screening awareness among the general population (5). Hypertension can lead to coronary artery disease, stroke, heart failure, kidney failure, and premature mortality (6), which are mostly preventable through low-cost medications and timely interventions.

Several factors contribute to this higher prevalence of hypertension in South Asia, including physical inactivity, decreased awareness, smoking, unhealthy diet, access to healthcare, cost of medications (5, 7–9). However, most of the studies lacked population representativeness, had a small sample size, and used a wide range of tools to measure risk factors. Several risk prediction models have been successfully used to identify and stratify patients according to their risk factors and initiate preventative therapies, for example, Framingham Risk Score for predicting coronary heart disease (10) and American College of Cardiology/American Heart Association (ACC/AHA) Pooled Cohort Equations Risk Calculator (11). However, these models have several limitations, including non-representative populations, inadequate ethnic diversity, selected endpoints, and poor reliability (11). There is a need to develop population-specific risk prediction models for people in South Asia.

In recent years, machine learning (ML) techniques have been shown to outperform traditional statistical approaches in developing risk stratification tools for diagnosing cardiovascular diseases (12–15). ML is a branch of computer science that broadly enables computers to “learn” without being directly programmed (13) and process large data with complex interactions. Although ML algorithms are not based on causal inference compared to traditional statistical methods, it is still a critical approach to estimating causal effects in observational studies. ML often shows superior performance compared to traditional ststistical techniques for reducing bias, automatic managing of missing variables with less manipulation of original data, controlling for confounding and data balancing- a key factor that leads to improved results (13). ML provides accuracy values, for example, 85% accuracy for a model suggest that the algorithm correctly identified 85 out of 100 participants which can aid in decision making. In addition, ML techniques excel in analyzing “big data” problems where commonly used statistical approaches struggle. Thus, ML methodologies can help develop automated tools for disease prediction, decision-aids, and identifying likely rates of hypertension in a population (13).

A recent review identified and examined ML techniques in hypertension detection and reported a a lack of studies combining sociodemographic and clinical data with signal processing which could increase model performance (16). A previous study used ML algorithms for automatic classification of hypertension using personal features but failed to include sociodemographic data (17). Another study in India developed ML risk stratification algorithms for diabetes and hypertension using data from 2,278 patients collected by community health workers (18). Two studies in China used ML to detect hypertension using electronic health records (19, 20). Despite the advancement in ML models for individual risk prediction for different diseases, no studies have used ML models to predict hypertension at a population level and validated the models using large datasets in South Asian countries. We, therefore, aimed to use ML approaches to identify factors associated with hypertension diagnosis and compared the model's performances to predict hypertension in three South Asian countries.

## Methods

### Study Design

A retrospective study using ML analyses to detect hypertension using cross-sectional nationally representative population-based surveys.

### Data Source and Variables

We obtained individual-level de-identified data from the most recent nationally representative and internationally comparable Demographic and Health Survey (DHS) in Bangladesh (2017–18), Nepal (2016), and India (2015–16). We created a single dataset by harmonizing data from each survey to a standard data specification. We included 818,603 respondents who provided consent for measurement of blood pressure, weight, height and had complete data for the analyses. Participants with incomplete data were excluded. The details on DHS survey design, data availability is available on the DHS website (https://www.dhsprogram.com) (21). In brief, the DHSs are periodic nationally representative household surveys that collect data for a wide range of variables on population, health, and nutrition. These surveys usually are conducted by a national implementing agency in collaboration with the Ministry of Health and technical assistance provided by ICF International USA and USAID. Participants in DHSs are generally selected using stratified two-stage cluster sampling. Firstly, sampling census enumeration areas are selected using probability proportional to size sampling technique through statistics provided by the respective national statistical bureaus. Secondly, the administrative wards at the community level are considered primary sampling units (PSUs). Following systematic random sampling, the households are selected from the sampled PSUs. Data are provided by the household head or a member who has detailed information about the household and family members. Subsamples of eligible participants are chosen for biomarker testing (e.g., height, weight, and blood pressure) (22). DHS surveys have a very high response rate, usually more than 90%. We used the household member record dataset, which has one record for every household member.

#### Blood Pressure Measurement and Hypertension

Blood pressure (BP) was measured for participants using the DHS standard protocol (23, 24). In brief, three measurements were taken by trained health workers, at seating position, at about 10 min intervals. The mean of the second and third measurements was used to record systolic BP and diastolic BP. We defined hypertension based on the cut-offs provided by the Seventh Report of Joint National Committee on Prevention, Detection, Evaluation, and Treatment of High Blood Pressure (JNC7) guideline (25) where an individual was categorized as hypertensive if they had systolic BP ≥ 140 mmHg or diastolic BP ≥ 90 mmHg or reported to use antihypertensive medication during the survey.

#### The Biomarker Questionnaire

The biomarker questionnaire collected details on height, weight, hemoglobin, BP, and random blood glucose for women aged 15–49 and men aged 15–54. The response rate for BP measurements was 97% among women and 92% among men. Furthermore, irrespective of their BP, all participants were asked, “Were you told on two or more different occasions by a doctor or other health professional that you had hypertension or high blood pressure?” If they responded in the affirmative, they were asked a follow-on question, “To lower your blood pressure, are you taking a prescribed medicine?”.

#### Other Covariates

DHS collected information on a wide range of variables from the selected households and the respondents from those households using face-to-face interviews conducted by trained personnel. Data on sociodemographic and economic factors like age, sex, education, and household wealth index were included. The education categories are defined based on the number of years of education completed by an individual: 0 year as “no education”; 1–5 years as “primary education”; 6–12 years as “secondary education”; and more than 12 years of educational attainment categorized as “higher studies”. For household wealth index, each national implementing agency constructed a country-specific index using principal components analysis from data on household assets including durable goods (i.e., bicycles, televisions, etc.,) and dwelling characteristics (i.e., sanitation, source of drinking water, and construction material of the house, etc.,) (14). This wealth index was then categorized into five groups (i.e., poorest, poorer, middle, richer, and richest) based on the quintile distribution of the sample.

### Data Analysis

Data were presented as mean ± SD for continuous variables and frequency (%) for categorical variables. We performed the chi-square test to assess the difference between hypertension and non-hypertension individuals for each categorical variable. *P*-value < 0.05 was considered significant. The wealth index was converted into a dichotomous variable; the bottom 60%, is, “poorest”, “poor” and “middle” were combined into one group (low SES). The remaining two categories were clubbed into the other category (high SES). The following risk factors of hypertension were included in all the ML models: age, BMI, education, wealth, systolic BP, diastolic BP, taking medicine for high BP, and ever told by a physician to take BP medications based on data availability. Data analyses were performed using SPSS version 24 and Matlab software.

We then experimented with the six most commonly used supervised ML models to measure their predictive effectiveness to diagnosis hypertension using a classification task. In this task, we included hypertension as a independent variable and the other factors as independent variables. After model training, we measured the accuracy, precision, recall f1 score, and log loss values. The higher accuracy, precision, recall, and f1-score suggested comparatively better models. On the other hand, lower log loss value represented a better model. All the evaluation matrices used were within the range 0–100. In addition, we included feature ranking (considering the coefficient values of the features) suggesting factors that were mostly contributed for hypertension.

#### Decision Tree (DT)

A DT algorithm can be used for both classification and regression on a given dataset. Each node of the tree represents a specific condition on one of the dataset attributes. The decision process starts from the tree root. Each node's condition is checked based on which node edges are chosen, and the decision process continues to the next tree level. This trend continues until a leaf node is reached based on which the final decision is presented (1). A high-information-gain parameter at a node can partition the training data to increase classification accuracy. Entropy (E) and information-gain (IG) is calculated as


(1) $$\begin{array}{r} E\left(Y\right)=\;-\sum_{k}\;p_{k}{\text{log}}_{2}\left(p_{k}\right), \end{array}$$

(2) $$\begin{array}{r} IG\left(D,\;X\right)=E\left(D\right)-\sum_{v\in Values\left(X\right)}\frac{\left|D_{v}\right|}{\left|D\right|}\;.E\left(D_{v}\right), \end{array}$$

where Y and X are variables, D is data, *p*_*k*_ is the probability of observing value k for variable Y.

#### Random Forest (RF)

RF employs multiple decision trees to improve classification and regression performance. RF creates bootstraps by random resampling from the training dataset, and in the end, it combines the results (2). Bootstrap aggregation is used during training in RF algorithms. After training, the model can predict output given an unseen sample x' by averaging the predictions performed by all decision trees:


(3) $$\begin{array}{r} -\bar{f}=\frac{1}{B}\sum_{b=1}^{B}\;f_{b}\left(x^{{\;}^{\prime }}\right) \end{array}$$

where B is the total number of decision trees of the random forest, $f_{b}\left(x^{\prime }\right)$ is the prediction of b-th decision tree give input *x*′.

#### Gradient Boosting Machine (GBM)

GBM is another fixed-size tree-based algorithm that also can be used for classification and regression. It uses boosting strategies for better performance (3).

#### Extreme Gradient Boosting (XGBoost)

XGBoost is another tree-based algorithm that uses a gradient boosting framework. This algorithm can solve large-scale real-world problems using comparatively fewer resources than GBM (4).

#### Logistic Regression (LR)

LR is one of the classification methods in ML which uses logistic functions for binary dependent variables (5). The generalized fundamental linear equation for LR is,


(4) $$\begin{array}{r} g\left(E\left(y\right)\right)=\alpha +\beta x_{1}+\gamma x_{2} \end{array}$$

where the link function is denoted as g (.), the target variables expectation is denoted as E(.), and the right side of the equation is the linear prediction.

#### Linear Discriminant Analysis (LDA)

LDA is used in machine learning, statistics, and pattern recognition problems. LDA is similar to regression analysis and analysis of variance (ANOVA) (5).

#### Model Evaluation

We compared the performance of the ML-classifiers using accuracy, precision, recall, F-1 score, and log loss, respectively. Using 10-fold cross-validation, the whole dataset was split into 10 subsets. In each fold, one of the subsets was used for model testing and the remaining subsets for model training. The training/testing process is repeated 10 times, corresponding to 10 folds. The evaluation metrics are calculated using the following equations:


(5) $$\begin{array}{r} Precision=\frac{TP}{TP+FP}, \end{array}$$

(6) $$\begin{array}{r} Recall=\;\frac{TP}{TP+FN}, \end{array}$$

(7) $$\begin{array}{r} F_{1}=2\;\times \frac{Precision\;\times Recall}{Precision+Recall} \end{array}$$

(8) $$\begin{array}{r} H_{p}\left(q\right)=\;-\;\frac{1}{N}\sum_{i=1}^{N}y_{i}.\text{log}\left(p\left(y_{i}\right)\right) \end{array}$$

(9) $$\begin{array}{r} +\left(1-\;y_{i}\right).\text{log}\left(1-p\left(y_{i}\right)\right) \end{array}$$

where TP is true positive rate, FP is false positive rate, FN is false-negative rate, y is target variable level, p (y) is predicted probability and *H*_*p*_(*q*) is log-loss.

#### Ethics Approval

DHS surveys received ethical approval from the ICF Institutional Review Board and country-specific review boards. Informed consent was taken from each participant to participate in the study. The DHS program authorized researchers to use relevant datasets for analysis upon submission of a brief research proposal. The data used in this study were anonymized to protect privacy, anonymity, and confidentiality. Therefore, no ethics approval is required for this research. More details on survey design, ethical approval, data availability can be found on the DHS program website (https://dhsprogram.com/).

## Results

Of the 8,18,603 participants included in the analyses, 82,748 (overall 10.11%) had hypertension. The majority of the participants were from the 15–24 years age group (33.68%), followed by the 25–34 years age group (28.90%), 35–44 years age group (23.85%), and 45 + years age group (13.57%). The majority of the participants (58.60%) had normal BMI, while 21.78% were underweight, 14.95% were overweight, and 4.67% were obese. Nearly half of the study participants received secondary education (47.43%) and were from a low-income family (44.91%). More than 90% of the respondents were informed about their high BP by a doctor, and 96.82% reported taking prescribed medicine to lower BP. However, only 60.28% measured their BP regularly. Participants' characteristics and significant variables are listed in Table 1.

**Table 1:** Background characteristics of the participants (*N* = 8,18,603).

**Variables**	**Total**	**Level of hypertension**		***p*-value**
		**No-hypertension**	**Hypertensive**	
	***n*** **(%)**	***n*** **(%)**	***n*** **(%)**	
**Age of the respondent**				
15–24	2,75,719 (33.68)	2,67,605 (97.06)	8,114 (2.94)	*p* < 0.001
25–34	2,36,583 (28.90)	2,18,453 (92.34)	18,130 (7.66)	
35–44	1,95,200 (23.85)	1,64,615 (84.33)	30,585 (15.67)	
45+	1,11,102 (13.57)	85,183 (76.67)	25,919 (23.33)	
Total	8,18,604 (100.00)	7,35,856 (89.89)	82,748 (10.11)	
**Level of BMI**				
Thin	1,78,284 (21.78)	1,69,855 (95.27)	8,429 (4.73)	*p* < 0.001
Normal	4,79,687 (58.60)	4,37,626 (91.23)	42,061 (8.77)	
Overweight	1,22,414 (14.95)	99,659 (81.41)	22,755 (18.59)	
Obese	38,218 (4.67)	28,715 (75.13)	9,503 (24.86)	
Total	8,18,603 (100.00)	7,35,855 (89.89)	82,748 (10.11)	
**Level of education**				
No education	2,12,323 (25.94)	1,84,683 (86.98)	27,640 (13.02)	*p* < 0.001
Primary	1,12,656 (13.76)	99,095 (87.96)	13,561 (12.04)	
Secondary	3,88,283 (47.43)	3,55,180 (91.47)	33,103 (8.53)	
Higher	1,05,342 (12.87)	96,898 (91.98)	8,444 (8.02)	
Total	8,18,604 (100.00)	7,35,856 (89.89)	82,748 (10.11)	
**Wealth status**				
Poor	3,67,661 (44.91)	3,38,073 (91.95)	29,588 (8.05)	*p* < 0.001
Middle	1,62,088 (19.80)	1,45,454 (89.74)	16,634 (10.26)	
Rich	2,88,854 (35.29)	2,52,329 (87.36)	36,525 (12.64)	
Total	8,18,603 (100.00)	7,35,856 (89.89)	82,747 (10.11)	
**Ever measured blood pressure**				
No	3,14,579 (39.72)	2,88,775 (91.80)	25,804 (8.20)	*p* < 0.001
Yes	4,77,383 (60.28)	4,24,845 (88.99)	52,538 (11.01)	
Total	7,91,962 (100.00)	7,13,620 (90.11)	78,342 (9.89)	
**Told by a doctor to have high blood pressure**				
No	7,22,313 (91.21)	6,53,345 (90.45)	68,968 (9.55)	*p* < 0.001
Yes	69,648 (8.79)	60,275 (86.54)	9,373 (13.46)	
Total	7,91,961 (100.00)	7,13,620 (90.11)	78,341 (9.89)	
**Taking prescribed medicine to lower blood pressure**				
No	7,66,754 (96.82)	6,92,265 (90.29)	74,489 (9.71)	*p* < 0.001
Yes	25,208 (3.18)	21,356 (84.72)	3,852 (15.28)	
Total	7,91,962 (100.00)	7,13,621 (90.11)	78,341 (9.89)	

Participants in the higher age groups (>45 years) were more likely to be hypertensive, compared to the younger age groups (24 years) (23.33 vs. 2.94%). Participants with no education were more likely to be hypertensive (13.02%) than those with higher education (8.02%). Further, participants in the rich wealth index were more likely to be hypertensive compared to the poor wealth index (12.64 vs. 8.05%). All of these differences were statistically significant (*p*-value < 0.001). The prevalence of hypertension was 11.01% among respondents who ever measured BP and 13.46% among those who had high BP diagnosed by a doctor. 15.28% of the respondents taking medication for BP had hypertension. Results of the bivariate analysis show that all the sociodemographic variables had a statistically significant relationship with hypertension (*P* < 0.05) (Table 1).

**Figure 1:**
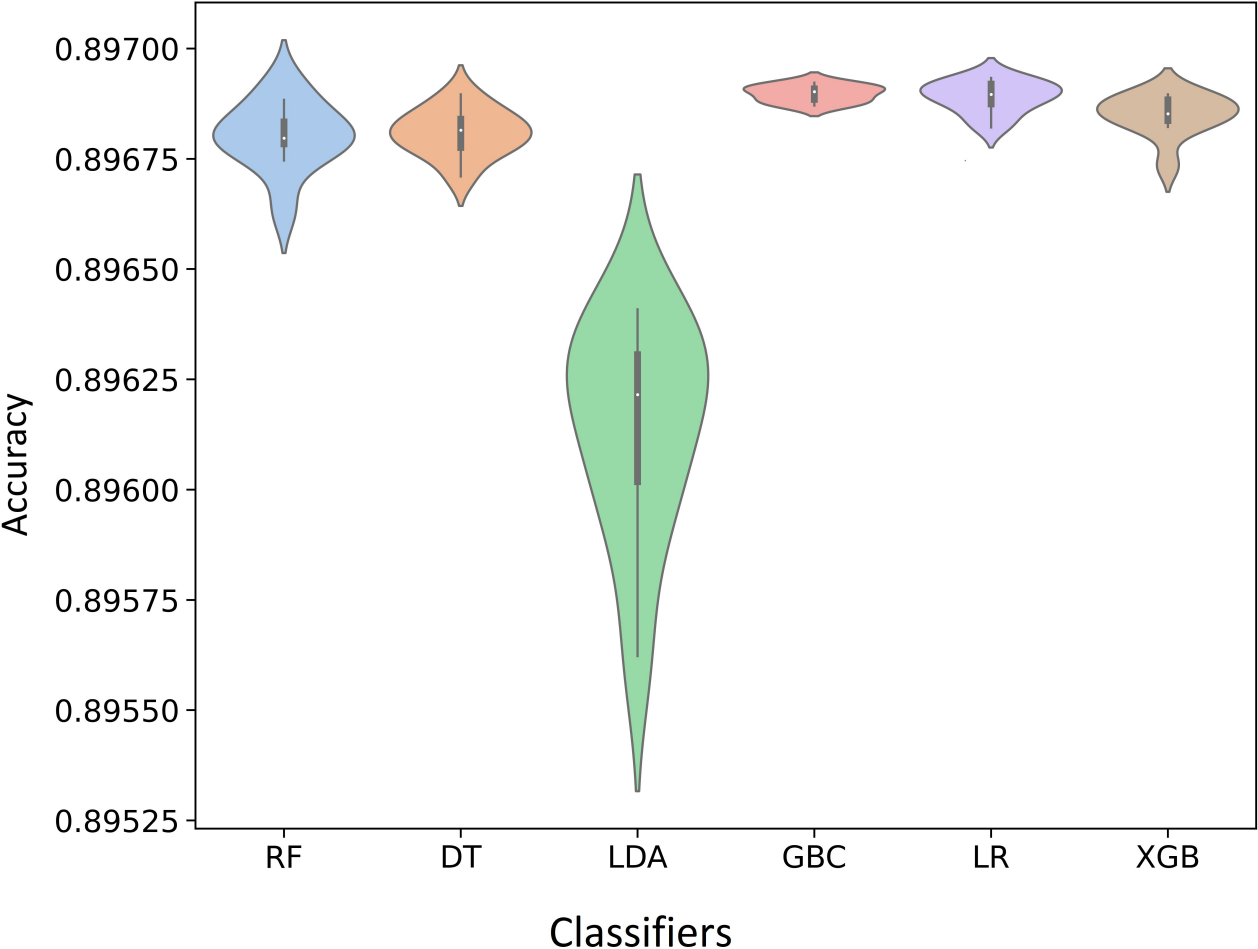
Violin plot of the 10-fold cross-validation (Violin plots representing the entire distribution).

**Figure 2:**
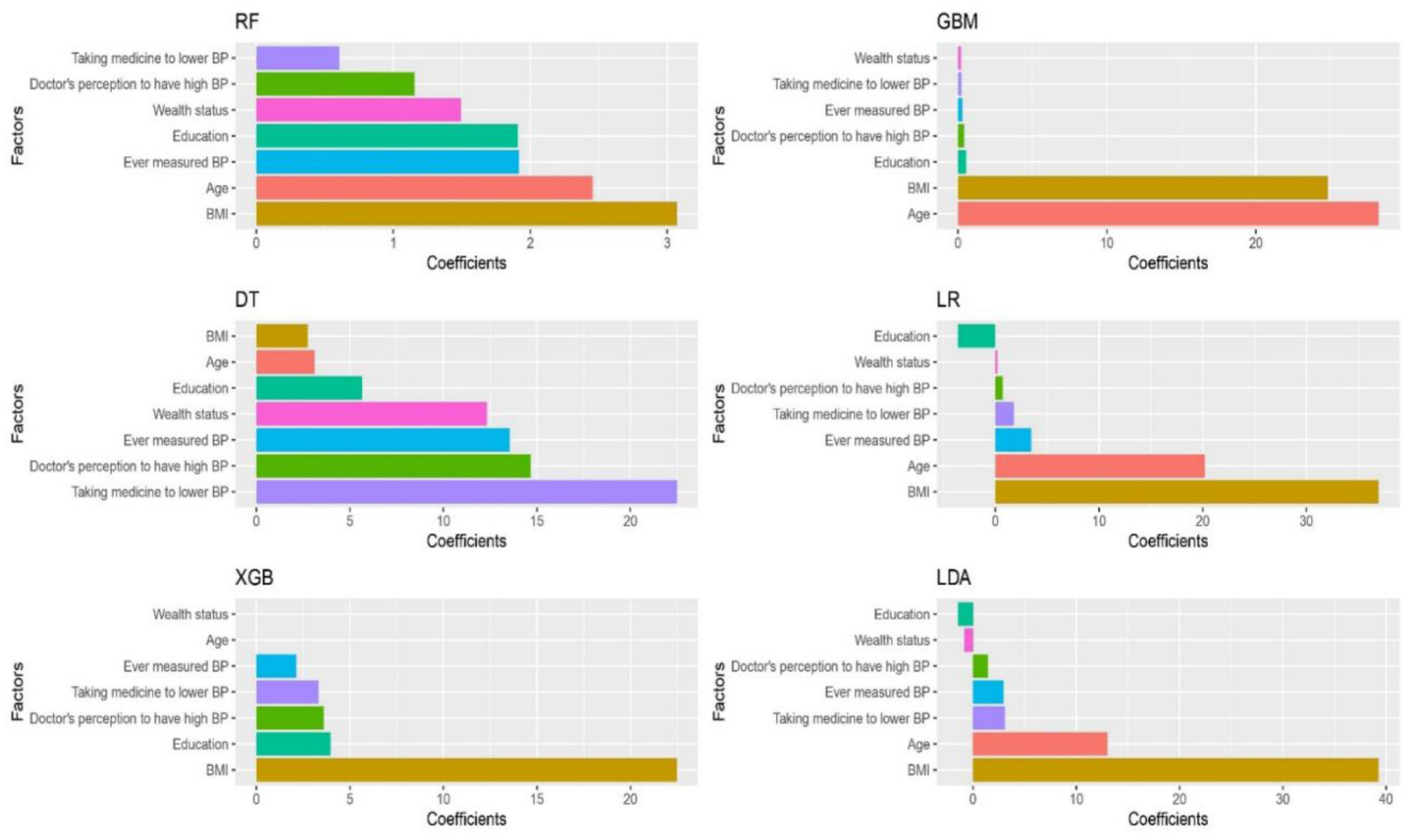
Significant features for hypertension in three South Asian countries.

According to performance metrics presented in Table 2, all the algorithms performed with a reasonable accuracy score (>80%). XGBoost, GBM, LR, and LDA achieved the highest accuracy of 90%, while RF and DT achieved 89 and 83%, respectively. DT reached the precision value of 91%, and the rest performed with 90%. XGBoost, GBM, LR, and LDA achieved the highest recall value, 100%, while RF scored 99% and DT scored 90%. Regarding the F1-score, XGBoost, GBM, LR, and LDA scored 95%, the highest, while RF scored 94%, and DT scored 90%. All the algorithms performed with good and small log loss values for the last evaluation criteria log loss values <6%. Figure 1 shows that GBM provided the highest mean accuracy, followed by LR and XGBoost (XGB in Figure 1). Unlike the boxplot, the entire distribution of the 10-fold accuracy can be visualized in the violin plot (Figure 1). The significant features determined by the algorithms after training are shown in Figure 2. Most of the algorithms found that the significant factors for hypertension were age and BMI. Ever measured BP, education, taking medicine to lower BP, and doctor's perception of high BP was also significant but comparatively lower than age and BMI (Figure 2).

**Table 2:** Performance indicators of all selected machine learning algorithms.

**Algorithms**	**Accuracy**	**Precision**	**Recall**	**F1 score**	**Log loss**
Random forest	0.89	0.90	0.99	0.94	3.63
Decision tree	0.83	0.91	0.90	0.90	5.92
XGB	0.90	0.90	1.00	0.95	3.52
GBM	0.90	0.90	1.00	0.95	3.33
LR	0.90	0.90	1.00	0.95	3.55
LDA	0.90	0.90	1.00	0.95	3.57

*XGB, XGBoost; GBM, Gradient Boosting Machine; LR, Logistic Regression; LDA, Linear Discriminant Analysis*.

## Discussion

To our knowledge, this is the first study to apply ML approaches to predict hypertension and its associated factors using population-representative data in three South Asia countries. We identified seven risk factors associated with hypertension in the South Asian population: age, BMI, education, wealth status, ever measuring BP, being diagnosed by a doctor, and taking medication to lower BP. After applying ML algorithms, we observed that XGBoost, GBM, LR, and LDA had the highest accuracy and recall with a score of 90 and 100%, respectively. DT achieved the highest precision value of 91%. ML models are superior to traditional statistical techniques where complex relationships between variables may not be fully explained using standard statistics. Our work has implications for hypertension prevention by applying these ML models to population-level data for hypertension screening among the population and automating the tasks without substantial human labor.

Several recent studies have proposed ML models to predict hypertension using a variety of demographic, biomarker, fitness, and spirometry data in various combinations (26–28). Ture et al. used age, gender, smoking, lipoprotein, and triglyceride levels and evaluated the performance of three three decision trees, four statistical algorithms, and two neural networks on 694 participants (29). Radial Basis Function showed the best performance for sensitivity (95.24%), specificity (66.67%), and predictive rate (81.48%). Similarly, Heo and colleagues developed hypertension prediction ML models using obesity, biomarkers, and spirometry data. They identified that obesity indices were most closely related to the risk of developing hypertension where wrapper-based feature selection methods-LR model showed the best performance (sensitivity and specificity of 0.813 and 0.401, respectively (30). A study in Canada using medical records and demographics used a neural network model for predicting hypertension with about 82% accuracy (26).

Several recent studies have demonstrated that ML models might be feasible and valuable for predicting and managing hypertension (27, 31). However, despite the growing interest, ML-informed BP prediction is yet to be implemented in clinical practice due to limitations such as lack of ML algorithms, consistency, accuracy, and reliability (31). Recently, a study in Korea utilized three different classification methods, namely LG, LDA, and classification and regression tree for hypertension risk prediction using an extensive database. All three methods performed reasonably well, with LR only marginally better with 58% accuracy (32). A similar study applied ML algorithms on patients with hypertension using 12-year longitudinal data from a nationwide cohort with 55 variables, where RF showed the best performance (F1-score = 0.772) in terms of generalization to detect high-risk patients (33).

Our study found BMI to be a good predictor of hypertension. Previous studies have shown obesity is strongly related to the risk of developing hypertension (34–36). Studies in the United States, China, and India have shown that BMI is a risk factor for hypertension (37–39), whereas waist circumference is correlated with cardiovascular diseases (34, 38). Another study in China using ML identified high education, sedentary job, a positive family history, overweight, physical activity, and unhealthy diets as risk factors for hypertension (40). A prospective cohort of 33,000 people from India, Pakistan, and Bangladesh reported a significantly higher prevalence of hypertension among urban-dwelling, higher education, and higher wealth index participants (41). We found a significant association of hypertension with BMI, wealth status, and education in the South Asian population. These disparities might be due to lack of access to healthcare facilities, poor BP screening, recording, reporting, lack of awareness regarding risk factors, and inappropriate treatment (42, 43).

Several mathematical techniques and ML models have been used to develop risk prediction models in healthcare (44, 45). Ture and colleagues' used DTs, statistical algorithms, and neural networks and identified that neural networks have the best predictive ability for hypertension using risk factors as inputs (30). However, a limitation to this study was the missing obesity data which is known to be associated with hypertension (29). Heo and colleagues also developed hypertension prediction models using DT, LR, and NB classifiers using obesity, biomarkers, and spirometry indices as variables in creating these models. An important limitation of this study is the lack of data on wealth index, education levels, smoking, alcohol use, and physical activity (30). We utilized non-invasive data to develop ML models to predict hypertension in the South Asian population. Previous studies in South Asia have shown the effectiveness and cost-effectiveness of mobile phones and digital technologies (46–50). However, there is a need for demographic representativeness in training data, model transparency and standardized frameworks for using these ML prediction models to improve representativeness and reproducibility (51).

The following limitations should be considered when interpreting the findings. First, a limited number of variables are included in the models. Data on risk factors such as family history, race, alcohol consumption, waist-hip ratio, physical activity levels, dietary intake, and biochemical parameters (e.g., blood glucose, lipid profile) were unavailable for all countries, which might have affected the measurement precision of our models. Second, the risk factors may have changed since some study data were from the 2016 survey. Third, ML models have an inherent weakness in making claims about causation. Finally, we could not externally validate our models using other data sources from these countries. Therefore, our results should be interpreted with caution. Despite these limitations, the primary strength of this study is the use of large-scale nationally representative survey data from 3 South Asian countries using ML approaches to predict hypertension.

Future research on developing country-specific risk assessment tools and validation are essential since risk factors, particularly demographics, education level, and wealth index, are not the same among different countries. These models can also be made available online or via mobile phone applications where individuals can check their risks of developing hypertension at home by answering simple questions such as their age, BMI, and sex. Models based on robust biochemical data, electronic health records (52) and external validation are recommended in the future A two-stage approach can be put into clinical practice, where ML model identifies individuals who are at risk of hypertension and in the second stage, at-risk individuals undergo evaluation by a physician for a confirmed diagnosis and appropriate treatment (53).

## Conclusion

Our study suggests that using simple, non-invasive information, ML models can predict hypertension among the South Asian population with high accuracy. Age and BMI were the most significant risk factors associated with hypertension in our study population. Further research is needed to include other risk factors and biomarkers associated with hypertension. ML models can then be trained and incorporated into the dataset to develop population-based hypertension risk assessment tools.

## Data Availability Statement

Publicly available datasets were analyzed in this study. This data can be found here: data are available from DHS website upon registration and request.

## Ethics Statement

The studies involving human participants were reviewed and approved by the Institutional Review Boards (IRBs) of the ICF International, Rockville, Maryland, USA and Respective Country National Ethics Board approved the study. The permission for using the data was obtained in December 2019. All participants provided informed consent to participate into the survey. The patients/participants provided their written informed consent to participate in this study.

## Author Contributions

SI: concept, study protocol development, drafting, and preparing the final manuscript for submission. AT, MMA, BA, and MAA: data analysis and drafting. All authors have contributed to intellectual inputs and review of the manuscript.

## Funding

SI is funded by the National Heart Foundation of Australia (102112) and a National Health and Medical Research Council (NHMRC) Emerging Leadership Fellowship (APP1195406).

## Conflict of Interest

The authors declare that the research was conducted in the absence of any commercial or financial relationships that could be construed as a potential conflict of interest.

## Publisher's Note

All claims expressed in this article are solely those of the authors and do not necessarily represent those of their affiliated organizations, or those of the publisher, the editors and the reviewers. Any product that may be evaluated in this article, or claim that may be made by its manufacturer, is not guaranteed or endorsed by the publisher.
